# The precision and agreement of corneal thickness and keratometry measurements with SS-OCT versus Scheimpflug imaging

**DOI:** 10.1186/s40662-020-00197-0

**Published:** 2020-06-09

**Authors:** Yune Zhao, Ding Chen, Giacomo Savini, Qing Wang, Hongfang Zhang, Yili Jin, Benhao Song, Rui Ning, Jinhai Huang, Chenyang Mei

**Affiliations:** 1 School of Ophthalmology and Optometry and Eye Hospital, Wenzhou Medical University, Wenzhou, Zhejiang China; 2 Key Laboratory of Vision Science, Ministry of Health P.R. China, Wenzhou, Zhejiang China; 3 G.B. Bietti Foundation IRCCS, Rome, Italy; 4 Eye Hospital of Wenzhou Medical University, 270 West Xueyuan Road, Wenzhou, 325027 Zhejiang China

**Keywords:** Central corneal thickness, Children, Precision, Paracentral corneal thickness, Peripheral corneal thickness, Keratometry, Swept-source optical coherence tomography, Scheimpflug imaging

## Abstract

**Purpose:**

To assess the repeatability and reproducibility of swept-source optical coherence tomography (SS-OCT) and Scheimpflug system and evaluate the agreement between the two systems in measuring multiple corneal regions in children.

**Methods:**

Pachymetric and keratometric maps for both systems were evaluated. Central, midperipheral and peripheral corneal thickness (CT), keratometry and astigmatism power vectors were recorded. The three outcomes yielded by the same observer were used to assess intraobserver repeatability. The differences in the mean values provided by each observer were used to evaluate interobserver reproducibility. Within-subject standard deviation, test-retest repeatability (TRT) and coefficient of variation (CoV) were used to analyze the intraobserver repeatability and interobserver reproducibility. Paired T-test and Bland-Altman were used to appraise interdevice agreement.

**Results:**

Seventy-eight eyes of 78 children were included. The CoV was ≤2.12 and 1.10%, respectively, for repeatability and reproducibility. TRT and CoV were lower for central and paracentral CT measurements than for peripheral measurements. The SS-OCT device generated higher precision when acquiring CT data, whereas Scheimpflug system showed higher reliability when measuring corneal keratometry. Although the CT readings measured using SS-OCT were significantly thinner than Scheimpflug device (*P* <  0.001), the central and thinnest CT values were still of high agreement. The interdevice agreement of keratometry measurement was high for the central corneal region and moderate for the paracentral and peripheral areas.

**Conclusions:**

The precision of CT measurements by SS-OCT was higher, while the reliability of keratometry measurements by the Scheimpflug system was higher in children. Apart from the measured values in the central corneal region, the thickness and keratometry readings should not be considered interchangeable between the two systems.

## Background

Precise measurement of corneal thickness (CT) and refractive power in children is vital for screening corneal ectasia, monitoring myopia progression, and planning orthokeratology [1, 2]. The measurements are important as it not only includes the central cornea, but also the peripheral zone, and alterations in these could indicate the development of corneal diseases, such as keratoconus and Fuchs’ endothelial dystrophy [3, 4].

To obtain a topographic map of the cornea, various technologies including Placido disk corneal topography, slit-scanning corneal topography, Scheimpflug imaging and optical coherence tomography (OCT) have been employed. Placido disk imaging does not provide information regarding the posterior corneal surface. Slit-scanning generates a lower repeatability in characterizing the posterior corneal surface when compared with the Scheimpflug principle [5].

Several reports have revealed high precision of rotating Scheimpflug camera, the Pentacam (Oculus Optikgeräte GmbH, Wetzlar, Germany), and an anterior-segment OCT (AS-OCT), the CASIA SS-1000 (Tomey, Nagoya, Japan) in measuring the central corneal thickness (CCT) and power [5–9]. OCT is considered a high-resolution, real-time ocular imaging technology. Time-domain OCT combined with Placido disk corneal topographer [10–13], spectral-domain OCT (SD-OCT) with or without Placido disk imaging [14–16], and swept-source OCT (SS-OCT) were commercially released for acquiring the topographic map of cornea [17, 18]. Several studies have reported high precision of anterior segment SS-OCT, CASIA (SS-1000; Tomey, Nagoya, Japan), in acquiring pachymetric and keratometric data of the central cornea [17–19]. However, there is no study till date that has investigated the precision of these devices in measuring peripheral cornea under similar conditions. Additionally, there are no published papers that measured corneal topography in children, and its extent of cooperation remained low, challenging the reliability of measurement.

Thus, the purpose of this study was to comprehensively assess the intraobserver repeatability and interobserver reproducibility of the above-mentioned Scheimpflug camera and SS-OCT as well as to evaluate the interdevice agreement when measuring multiple corneal regions in children with myopia.

## Methods

### Subjects

This prospective study was conducted at the Eye Hospital of Wenzhou Medical University. The research protocol adhered to the tenets of the Declaration of Helsinki, and was approved by the Office of Research Ethics, Wenzhou Medical University (KYK2013–21). Signed informed consent forms by the guardians of subjects were obtained before undergoing examinations.

The exclusion criteria included children with trauma, acute ocular inflammation, any history of contact lens wear, previous ophthalmological surgeries, and ocular diseases other than ametropia. Before being enrolled in this study, all subjects underwent a complete ophthalmic examination, including subjective refraction, ophthalmoscopy, noncontact tonometry (TX-F; Cannon, Tokyo, Japan), slit-lamp microscopy and fundoscopy.

### Instruments

CASIA is an anterior segment SS-OCT device that uses a 1310 nm light source and produces a scan range with 6.0 mm depth and 16.0 mm diameter, yielding an axial resolution of ≤10 μm and a lateral resolution of ≤30 μm. The “Corneal Map” mode takes 0.3 s to obtain 16 radial B-scans at a range of 10 mm centered on the apical cornea, and each B-scan comprises of 512 A-scans. The captured information was then processed to generate the topographic map of the cornea.

The Pentacam HR is a high-resolution imaging system that works on the principle of Scheimpflug. It uses a slit-light source operating in a monochromatic blue light at a wavelength of 475 nm, and a 1.45-megapixel Scheimpflug camera rotating on the visual axis for taking 25 or 50 cross-section pictures of the anterior segment. In 2 s, up to 138,000 true elevation points are acquired to construct the corneal topography. The 25-picture scan mode was used in this study.

### Measurement procedures

In order to promote children’s compliance, one observer provided detailed instructions to each subject before beginning the measurement, and additionally the parent demonstrated to the child on how to cooperate during the examination. The subject was seated in a dim room with the chin on the chinrest and forehead against the forehead bar and was asked to fixate on the specified fixation point with both eyes wide open. Each device was manipulated according to the user’s manual. The scanning by Pentacam HR was automatically initiated when the corneal vertex was centered and focused manually, whereas the CASIA measurement was triggered manually after the alignment procedure was automatically accomplished by the system. To assure the measurement independence, patients were asked to move their head away from the chinrest, and the scan units were thoroughly retreated before subsequent examinations. The Pentacam data were considered valid if the “QS” index of the measurement showed “OK”. As for CASIA, the B-scan images were reviewed by the two observers individually after each measurement, ensuring that there was no apparent image artifact for OCT images. In addition, the observers also carefully performed checks for corneal maps to verify the scan quality.

Three successive scans were performed by each observer (DC and HFZ) between 9 AM and 5 PM. The observers were trained to use the device 1 month before this study began. The sequence of the 2 devices and the 2 examiners were randomly set. The time of whole measurement process for each subject was rigorously controlled within 20 min. The three measurements with each system were used to assess intraobserver repeatability. The outcomes of the 3 consecutive measurements obtained by the same observer were averaged, and the differences between the observers were used to evaluate interobserver reproducibility. The disparities regarding the parameters measured using CASIA and Pentacam HR were used to appraise the interdevice agreement.

**Fig. 1:**
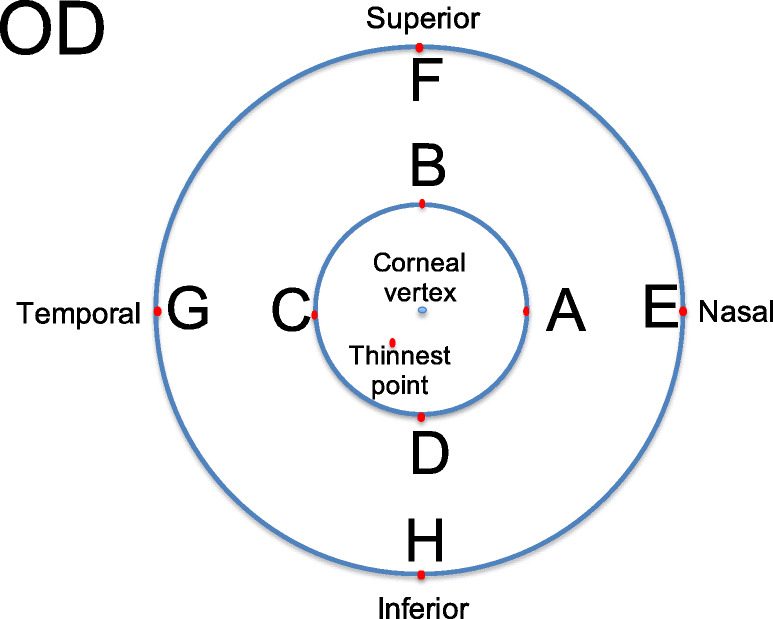
Paracentral and peripheral corneal indices measurements: Point A at 2-mm diameter circle nasally, Point B 2-mm superiorly, Point C 2-mm temporally, Point D 2-mm inferiorly, Point E at 5-mm diameter circle nasally, Point F 5-mm superiorly, Point G 5-mm temporally and Point H 5-mm inferiorly

Parameters were recorded on the following three zones:

1. The central zone of the cornea: CT values measured by each device included central corneal thickness (CCT) and thinnest corneal thickness (TCT); corneal power indices including the mean keratometry (K_m_) along with the steepest and the flattest anterior corneal meridians, and the magnitude and axis of astigmatism were analyzed using power vectors method (J_0_ and J_45_) as described by Thibos et al. [20]
2. The paracentral zone of cornea: The CT and keratometry at 2 mm diameter in the nasal (CT_2mm-Nasal_, K_2mm-Nasal_), superior (CT_2mm-Superior_, K_2mm-Superior_), temporal (CT_2mm-Temporal_, K_2mm-Temporal_) and inferior (CT_2mm-Inferior_, K_2mm-Inferior_) regions centered on the corneal vertex (Fig. 1).
3. The peripheral zone of cornea: The CT and keratometry at 5 mm diameter in the nasal (CT_5mm-Nasal_, K_5mm-Nasal_), superior (CT_5mm-Superior_, K_5mm-Superior_), temporal (CT_5mm-Temporal_, K_5mm-Temporal_) and inferior (CT_5mm-Inferior_, K_5mm-Inferior_) regions centered on the corneal vertex (Fig. 1).

### Statistical analysis

Statistical analyses were performed using SPSS (version 21.0, SPSS, IBM® Co, Armonk, New York, USA) and Microsoft Office Excel (Microsoft® Co, Redmond, Washington, D.C., USA). All data distributions were verified for normality by the Kolmogorov-Smirnov test. The results were expressed as mean ± standard deviation (SD). A *P* value of less than 0.05 was considered to be statistically significant.

To assess the intraobserver repeatability, one-way analysis of variance (one-way ANOVA) was performed for 3 consecutive measurements by each observer. Within-subject standard deviation (S_w_), test-retest repeatability (TRT), within-subject coefficient of variation (CoV), and intraclass correlation coefficients (ICC) were computed. Since astigmatism power vectors have small magnitudes (which make CoV quite large, so that it cannot represent the real variance among measurements) and can be either positive or negative, we did not rely on CoV to estimate their repeatability. Therefore, the precision for measurement of astigmatism power vector were assessed using ICC only. The TRT was calculated as 2.77 × S_w_, which was the expected upper limit for 95% of the difference between measurements [21]. The CoV was defined as 100% × S_w_ / overall means. An ICC higher than 0.9 was considered as high consistency, and an ICC between 0.75 to 0.90 was considered as moderately consistent, and an ICC less than 0.75 was considered as poor consistency [22]. To evaluate the interobserver reproducibility, the mean values obtained by the same observer were calculated, and S_w_, TRT, CoV, and ICC were computed for the 2 mean values obtained by the two observers. To appraise the interdevice agreement, paired T-test and Bland-Altman plots were applied, and 95% limits of agreement (LoA) was calculated as mean ± 1.96 SD of the differences between the two instruments.

## Results

A total of 78 right eyes from 78 children (47 males and 31 females) diagnosed with refractive errors were recruited. Among them, the proportion for 4 years old was 2.56%, for 5 years old was 3.85%, for 6 years old was 6.41%, for 7 years old was 16.67%, for 8 years old was 20.51%, for 9 years old was 19.23%, for 10 years old was 14.10%, for 11 years old was 10.26%, for 12 years old was 5.13%, and for 14 years old was 1.28%. The mean age of the subjects was 8.55 ± 1.97 years (range, 4–14 years), and the mean spherical equivalent refraction was − 1.49 ± 1.79 D (range, − 5.75 to 2.25 D).

### Intraobserver repeatability of corneal thickness measurements

For intraobserver repeatability of CT measurements by both devices, TRT and CoV were lower for CCT, TCT and paracentral CT measurements than for peripheral measurements. When taken individually, the TRT acquired with CASIA ranged from 2.98 to 12.42 μm, and the CoV was lower than 0.75% (Table 1). Both TRT and CoV were relatively higher for CT measurements with CASIA at superior locations of paracentral and peripheral cornea. Pentacam HR showed higher TRT and CoV on a scale of 14.68 to 34.19 μm and 0.98 to 2.12%, respectively (Table 2). The relatively greater TRT and CoV were also noticed when measuring the paracentral and peripheral cornea at inferior location. Comparison of the two instruments demonstrated that the TRT and CoV for CCT and TCT measurements generated by CASIA were lower than a quarter with respect to those provided by Pentacam HR. Regarding the paracentral and peripheral CT measurements, most of the TRTs and CoVs yielded by CASIA were about one-third of those rendered by Pentacam HR.

**Table 1:** Intraobserver repeatability outcomes for corneal thickness obtained using CASIA swept-source optical coherence tomography in children

Parameter	Observer	Mean ± SD	S_w_	TRT	CoV (%)	ICC (95% CI)
Center	1st	537.75 ± 29.16	1.15	3.18	0.21	0.998 (0.998 to 0.999)
	2nd	537.77 ± 29.10	1.08	2.98	0.20	0.999 (0.998 to 0.999)
Thinnest	1st	532.46 ± 29.67	1.45	4.02	0.27	0.998 (0.997 to 0.998)
	2nd	532.62 ± 29.56	1.50	4.15	0.28	0.997 (0.996 to 0.998)
Nasal 2 mm	1st	546.77 ± 29.00	1.95	5.41	0.36	0.995 (0.993 to 0.997)
	2nd	546.82 ± 29.08	1.77	4.91	0.32	0.996 (0.995 to 0.998)
Superior 2 mm	1st	549.15 ± 29.19	2.32	6.42	0.42	0.994 (0.991 to 0.996)
	2nd	548.98 ± 29.41	2.14	5.92	0.39	0.995 (0.992 to 0.996)
Temporal 2 mm	1st	536.99 ± 29.54	2.18	6.04	0.41	0.995 (0.992 to 0.996)
	2nd	536.92 ± 29.64	1.55	4.29	0.29	0.997 (0.996 to 0.998)
Inferior 2 mm	1st	537.42 ± 29.87	1.94	5.39	0.36	0.996 (0.994 to 0.997)
	2nd	537.65 ± 29.97	1.63	4.51	0.30	0.997 (0.996 to 0.998)
Nasal 5 mm	1st	586.02 ± 29.48	3.86	10.70	0.66	0.983 (0.975 to 0.989)
	2nd	586.26 ± 29.60	3.31	9.18	0.57	0.988 (0.982 to 0.992)
Superior 5 mm	1st	595.47 ± 30.30	3.96	10.97	0.67	0.983 (0.976 to 0.989)
	2nd	595.56 ± 31.10	4.48	12.42	0.75	0.980 (0.970 to 0.986)
Temporal 5 mm	1st	560.95 ± 30.29	3.53	9.77	0.63	0.987 (0.981 to 0.991)
	2nd	560.86 ± 30.40	2.74	7.59	0.49	0.992 (0.988 to 0.995)
Inferior 5 mm	1st	569.06 ± 31.43	3.65	10.11	0.64	0.987 (0.981 to 0.991)
	2nd	569.31 ± 31.37	3.23	8.96	0.57	0.989 (0.985 to 0.993)

Thickness data are in units of micrometer (μm); SD = standard deviation, S_w_ = within-subject standard deviation, *TRT* = test-retest repeatability (2.77 S_w_), *CoV* = within-subject coefficient of variation, *ICC* = intraclass correlation coefficient

**Table 2:** Intraobserver repeatability outcomes for corneal thickness obtained using Pentacam Scheimpflug imaging in children

Parameter	Observer	Mean ± SD	S_w_	TRT	CoV (%)	ICC (95% CI)
Center	1st	542.66 ± 27.90	6.17	17.09	1.14	0.953 (0.932 to 0.968)
	2nd	543.11 ± 28.85	5.30	14.68	0.98	0.967 (0.952 to 0.978)
Thinnest	1st	537.23 ± 28.42	7.27	20.15	1.35	0.937 (0.910 to 0.957)
	2nd	537.67 ± 29.06	5.97	16.54	1.11	0.959 (0.941 to 0.972)
Nasal 2 mm	1st	555.26 ± 28.14	6.79	18.82	1.22	0.944 (0.920 to 0.962)
	2nd	556.32 ± 29.41	6.11	16.92	1.10	0.958 (0.940 to 0.972)
Superior 2 mm	1st	562.38 ± 28.14	6.75	18.69	1.20	0.945 (0.921 to 0.962)
	2nd	562.66 ± 29.36	6.46	17.88	1.15	0.953 (0.933 to 0.968)
Temporal 2 mm	1st	542.44 ± 27.91	7.18	19.88	1.32	0.937 (0.909 to 0.957)
	2nd	542.77 ± 29.53	6.19	17.15	1.14	0.957 (0.939 to 0.971)
Inferior 2 mm	1st	543.71 ± 28.46	8.04	22.27	1.48	0.924 (0.892 to 0.948)
	2nd	544.04 ± 29.29	6.85	18.96	1.26	0.947 (0.924 to 0.964)
Nasal 5 mm	1st	610.17 ± 29.88	11.06	30.65	1.81	0.874 (0.824 to 0.914)
	2nd	611.59 ± 32.10	9.80	27.13	1.60	0.912 (0.876 to 0.940)
Superior 5 mm	1st	626.39 ± 31.49	11.51	31.89	1.84	0.877 (0.828 to 0.916)
	2nd	626.06 ± 32.73	10.94	30.30	1.75	0.896 (0.853 to 0.929)
Temporal 5 mm	1st	578.10 ± 29.13	11.00	30.46	1.90	0.870 (0.818 to 0.910)
	2nd	578.26 ± 31.92	10.32	28.59	1.78	0.902 (0.862 to 0.933)
Inferior 5 mm	1st	582.24 ± 31.00	12.34	34.19	2.12	0.857 (0.800 to 0.901)
	2nd	583.09 ± 31.86	11.72	32.46	2.01	0.876 (0.826 to 0.915)

Thickness data are in units of micrometer (μm); *SD* = standard deviation, *S*_*w*_ = within-subject standard deviation, *TRT* = test-retest repeatability (2.77 S_w_), *CoV* = within-subject coefficient of variation, *ICC* = intraclass correlation coefficient

### Intraobserver repeatability of corneal power measurements

With regards to the intraobserver repeatability of keratometry, the TRT and CoV in the central region were smaller than those in the paracentral and peripheral regions, and among these, the TRT and CoV for K_5mm-superior_ measurements remained the highest. The TRT for CASIA ranged from 0.28 to 1.32 D, and CoVs were ≤ 1.10% (Table 3). The TRT and CoV for Pentacam HR were on a scale of 0.25 to 0.98 D and 0.21 to 0.81%, respectively (Table 4). In addition, the TRT and CoV for central keratometry measurements were comparable between the two systems but were slightly greater with CASIA than with Pentacam HR for the paracentral and peripheral regions.

**Table 3:** Intraobserver repeatability outcomes for corneal power obtained using CASIA swept-source optical coherence tomography in children

Parameter	Observer	Mean ± SD	S_w_	TRT	CoV (%)	ICC (95% CI)
Km	1st	43.34 ± 1.43	0.10	0.28	0.23	0.995 (0.993 to 0.997)
	2nd	43.34 ± 1.43	0.11	0.30	0.25	0.994 (0.992 to 0.996)
J_0_	1st	−0.70 ± 0.43	0.11	0.32	–	0.933 (0.904 to 0.954)
	2nd	−0.73 ± 0.46	0.13	0.35	–	0.930 (0.900 to 0.952)
J_45_	1st	0.00 ± 0.21	0.12	0.34	–	0.724 (0.630 to 0.804)
	2nd	0.01 ± 0.23	0.14	0.37	–	0.715 (0.618 to 0.797)
Nasal 2 mm	1st	42.57 ± 1.36	0.23	0.64	0.55	0.971 (0.958 to 0.981)
	2nd	42.54 ± 1.38	0.25	0.68	0.58	0.969 (0.955 to 0.979)
Superior 2 mm	1st	44.15 ± 1.62	0.26	0.72	0.59	0.975 (0.964 to 0.983)
	2nd	44.20 ± 1.65	0.36	1.00	0.82	0.953 (0.933 to 0.969)
Temporal 2 mm	1st	42.73 ± 1.37	0.25	0.70	0.59	0.967 (0.953 to 0.978)
	2nd	42.72 ± 1.38	0.24	0.68	0.57	0.969 (0.956 to 0.979)
Inferior 2 mm	1st	43.94 ± 1.62	0.31	0.85	0.70	0.965 (0.949 to 0.976)
	2nd	43.97 ± 1.65	0.25	0.70	0.58	0.977 (0.966 to 0.984)
Nasal 5 mm	1st	41.92 ± 1.38	0.25	0.69	0.59	0.968 (0.954 to 0.979)
	2nd	41.91 ± 1.39	0.29	0.80	0.69	0.958 (0.940 to 0.972)
Superior 5 mm	1st	43.49 ± 1.71	0.48	1.32	1.10	0.926 (0.894 to 0.949)
	2nd	43.51 ± 1.65	0.43	1.18	0.98	0.936 (0.909 to 0.957)
Temporal 5 mm	1st	42.48 ± 1.40	0.30	0.83	0.70	0.956 (0.937 to 0.970)
	2nd	42.47 ± 1.35	0.24	0.67	0.57	0.969 (0.955 to 0.979)
Inferior 5 mm	1st	43.47 ± 1.58	0.36	1.00	0.83	0.950 (0.928 to 0.966)
	2nd	43.49 ± 1.60	0.36	1.00	0.83	0.951 (0.929 to 0.967)

Keratometric data are in units of diopter (D); *SD* = standard deviation, *S*_*w*_ = within-subject standard deviation, *TRT* = test-retest repeatability (2.77 S_w_), *CoV* = within-subject coefficient of variation, *ICC* = intraclass correlation coefficient

**Table 4:** Intraobserver repeatability outcomes for corneal power obtained using Pentacam Scheimpflug imaging in children

Parameter	Observer	Mean ± SD	S_w_	TRT	CoV (%)	ICC (95% CI)
Km	1st	43.13 ± 1.44	0.09	0.26	0.21	0.996 (0.994 to 0.997)
	2nd	43.14 ± 1.44	0.09	0.26	0.22	0.996 (0.994 to 0.997)
J_0_	1st	−0.71 ± 0.45	0.09	0.25	–	0.962 (0.945 to 0.974)
	2nd	−0.70 ± 0.45	0.09	0.25	–	0.961 (0.944 to 0.974)
J_45_	1st	−0.03 ± 0.21	0.11	0.30	–	0.766 (0.682 to 0.835)
	2nd	−0.04 ± 0.22	0.10	0.27	–	0.832 (0.767 to 0.883)
Nasal 2 mm	1st	42.23 ± 1.40	0.16	0.43	0.37	0.988 (0.982 to 0.992)
	2nd	42.25 ± 1.40	0.16	0.43	0.37	0.988 (0.982 to 0.992)
Superior 2 mm	1st	43.84 ± 1.63	0.25	0.69	0.57	0.977 (0.967 to 0.984)
	2nd	43.86 ± 1.66	0.23	0.63	0.52	0.982 (0.973 to 0.988)
Temporal 2 mm	1st	42.73 ± 1.37	0.19	0.52	0.44	0.981 (0.973 to 0.987)
	2nd	42.74 ± 1.37	0.18	0.50	0.42	0.983 (0.975 to 0.988)
Inferior 2 mm	1st	43.79 ± 1.71	0.25	0.69	0.57	0.979 (0.970 to 0.986)
	2nd	43.77 ± 1.68	0.22	0.60	0.50	0.983 (0.976 to 0.989)
Nasal 5 mm	1st	41.94 ± 1.40	0.12	0.32	0.28	0.993 (0.990 to 0.995)
	2nd	41.96 ± 1.41	0.11	0.31	0.27	0.994 (0.991 to 0.996)
Superior 5 mm	1st	43.50 ± 1.58	0.35	0.98	0.81	0.952 (0.931 to 0.967)
	2nd	43.51 ± 1.61	0.23	0.63	0.53	0.980 (0.971 to 0.987)
Temporal 5 mm	1st	42.48 ± 1.33	0.11	0.29	0.25	0.994 (0.991 to 0.996)
	2nd	42.48 ± 1.34	0.10	0.29	0.24	0.994 (0.991 to 0.996)
Inferior 5 mm	1st	43.62 ± 1.56	0.17	0.46	0.38	0.989 (0.983 to 0.992)
	2nd	43.60 ± 1.54	0.14	0.38	0.32	0.992 (0.988 to 0.995)

Keratometric data are in units of diopter (D); *SD* = standard deviation, *S*_*w*_ = within-subject standard deviation, *TRT* = test-retest repeatability (2.77 S_w_), *CoV* = within-subject coefficient of variation, *ICC* = intraclass correlation coefficient

As shown in Tables 3 and 4, the J_0_ measurement showed high repeatability for both CASIA and Pentacam HR with ICCs > 0.9. However, the repeatability of J_45_ measurement remained poor for CASIA with an ICC <  0.75, and moderate for Pentacam HR with an ICC ranging from 0.766 to 0.832.

### Interobserver reproducibility of corneal thickness measurements

With regards to the interobserver reproducibility of CT measurements, the TRT and CoV were higher for the paracentral and peripheral zones than those for CCT and TCT measurements. The TRT and CoV generated by CASIA were lower than 7.85 μm and 0.48%, respectively (Supp Table 1), while Pentacam HR yielded higher TRT and CoV ranging from 10.01 to 21.08 μm and from 0.67 to 1.31%, respectively (Supp Table 2). In comparison, the CoVs for all locations with CASIA were approximately one-third of those obtained with Pentacam HR.

### Interobserver reproducibility of corneal power measurements

With regards to the interobserver reproducibility of keratometry, the TRT and CoV for central cornea measurement were smaller when compared to those for paracentral and peripheral areas, and the highest TRT and CoV were observed for K_5mm-superior_ measurements. The TRT and CoV for cornea measurements with CASIA ranged from 0.25 to 0.72 D and 0.21 to 0.60%, respectively (Supp Table 3). For Pentacam HR, the TRT and CoV were ≤ 0.41 D and 0.34%, respectively (Supp Table 4). Comparison showed that TRT and CoV at all locations rendered by CASIA were higher than those provided by Pentacam HR.

The ICC for J_0_ measurement with CASIA was > 0.9, but for J_45_ was only 0.784 (Supp Table 3). As for Pentacam HR, the ICC value obtained was > 0.9 for both J_0_ and J_45_ measurements (Supp Table 4).

### Interdevice agreement of corneal thickness measurements

**Fig. 2:**
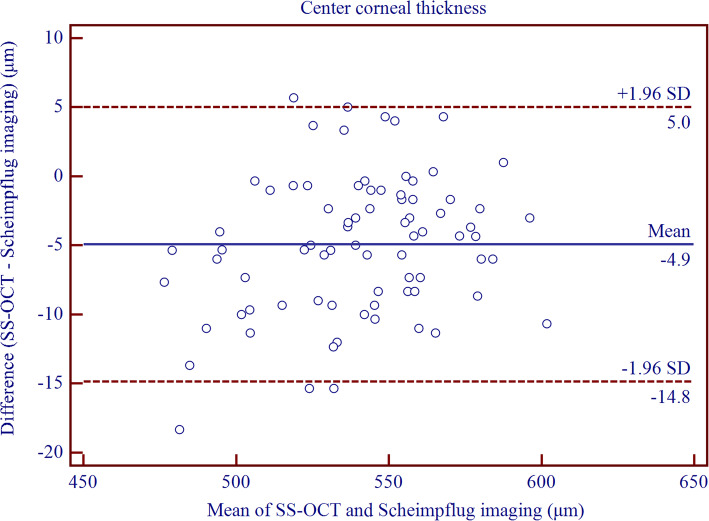
Bland–Altman plots showing pair-wise agreement between CASIA and Pentacam HR for central corneal thickness

**Fig. 3:**
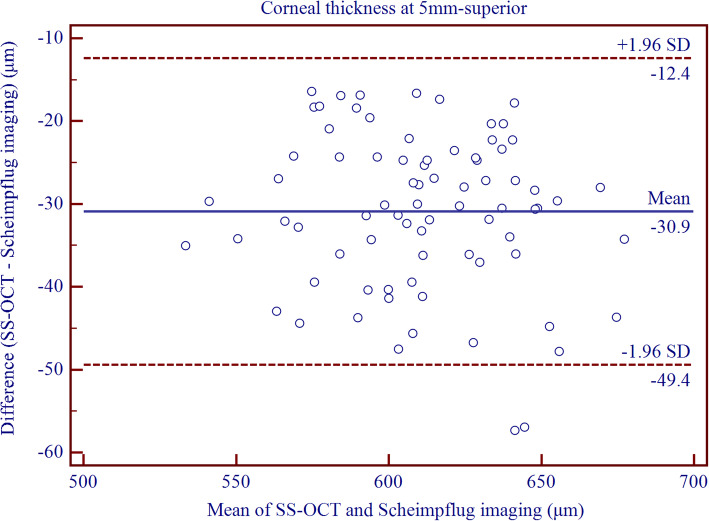
Bland–Altman plots showing pair-wise agreement between CASIA and Pentacam HR for superior corneal thickness at 5 mm diameter circle centered on the corneal vertex

Table 5 shows significantly thinner CT measurements with CASIA than with Pentacam HR (*P* <  0.0001 in all cases, paired T-test). The width of 95% LoA was the smallest for CCT and TCT measurements, and greater for peripheral CT acquirements (Fig. 2 and Fig. 3). For both the paracentral and peripheral CT measurements, relatively wider 95% LoAs were observed for the superior location.

**Table 5:** The difference and agreement for corneal thickness measurements between CASIA swept-source optical coherence tomography and Pentacam Scheimpflug imaging in children

Device Pairings	Mean ± SD	*P* Value	95% LoA
Center	−4.91 ± 5.06	< 0.001	−14.83 to 5.00
Thinnest	−4.77 ± 5.51	< 0.001	−15.56 to 6.03
Nasal 2 mm	−8.46 ± 5.59	< 0.001	−19.42 to 2.50
Superior 2 mm	−13.19 ± 5.73	< 0.001	−24.44 to − 1.95
Temporal 2 mm	−5.46 ± 5.80	< 0.001	−16.83 to 5.91
Inferior 2 mm	−6.30 ± 6.29	< 0.001	− 18.63 to 6.02
Nasal 5 mm	−24.16 ± 7.98	< 0.001	−39.80 to − 8.52
Superior 5 mm	−30.91 ± 9.45	< 0.001	−49.42 to − 12.39
Temporal 5 mm	−17.13 ± 7.78	< 0.001	−32.38 to −1.87
Inferior 5 mm	− 13.15 ± 9.52	< 0.001	−31.81 to 5.52

Thickness data are in units of micrometer (μm); Mean ± SD = Mean ± Standard deviation generated by paired T-test; 95% LoA = 95% limits of agreement

### Interdevice agreement of corneal power measurements

**Fig. 4:**
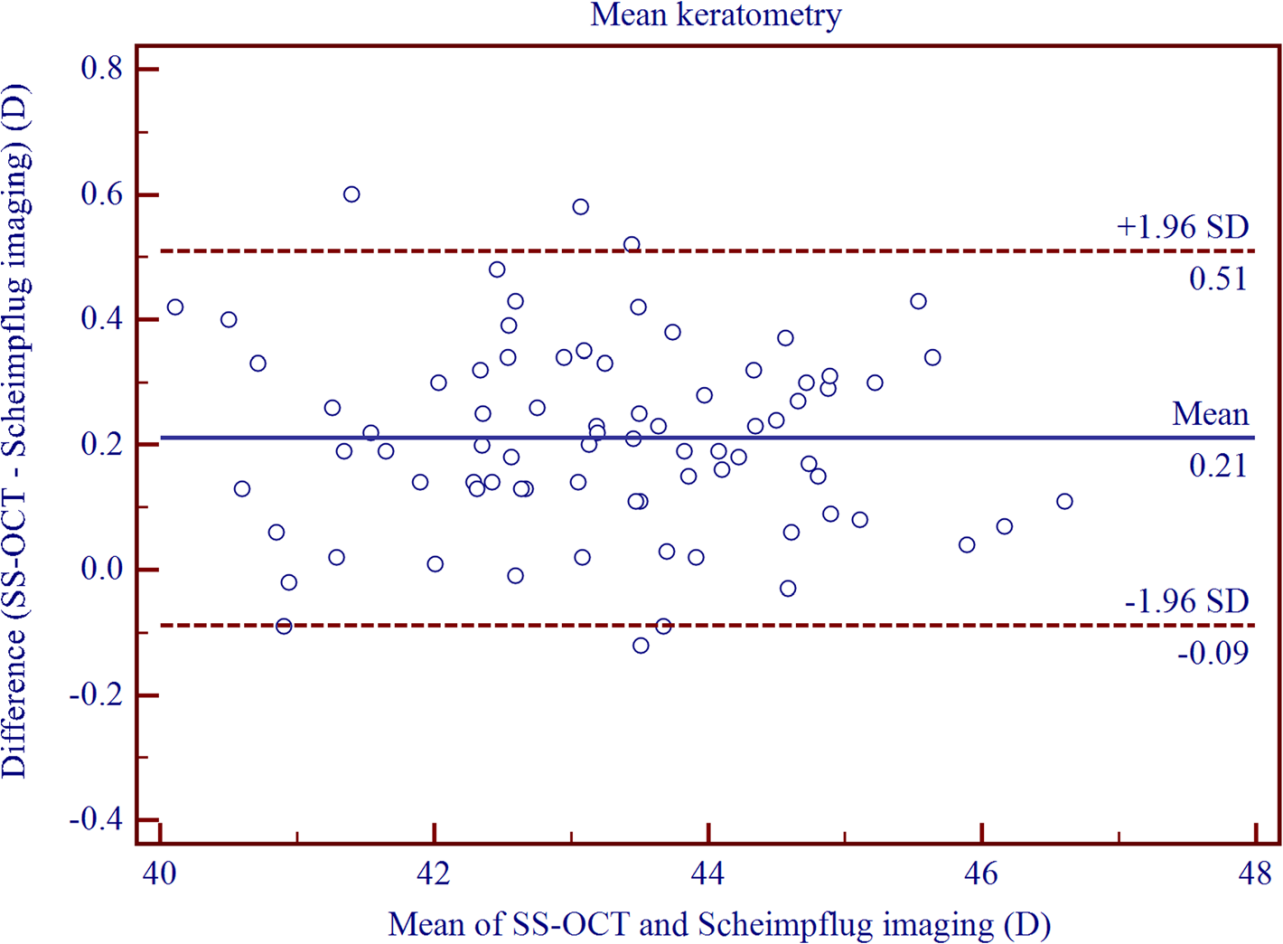
Bland–Altman plots showing pair-wise agreement between CASIA and Pentacam HR for mean of keratometry metrics (K_m_) along the steepest and the flattest anterior corneal meridians

**Fig. 5:**
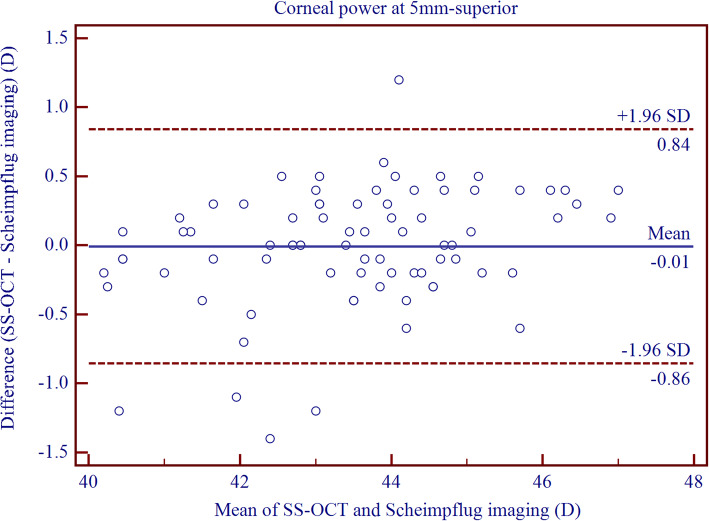
Bland–Altman plots showing pair-wise agreement between CASIA and Pentacam HR for superior corneal keratometry at 5 mm diameter circle centered on the corneal vertex

Table 6 shows the differences in keratometry measurements between CASIA and Pentacam HR (Fig. 4 and Fig. 5). The 95% LoA was narrowed for keratometry measurement in the central and temporal regions. Furthermore, the agreement of keratometry measurement for paracentral cornea was lower than that for the peripheral CT measurement.

**Table 6:** The difference and agreement for corneal power measurements between CASIA swept-source optical coherence tomography and Pentacam Scheimpflug imaging in children

Device Pairings	Mean ± SD	*P* Value	95% LoA
Km	0.21 ± 0.15	< 0.001	−0.09 to 0.51
J_0_	0.00 ± 0.12	0.849	− 0.23 to 0.23
J_45_	0.03 ± 0.15	0.116	− 0.26 to 0.31
Nasal 2 mm	0.33 ± 0.27	< 0.001	− 0.21 to 0.87
Superior 2 mm	0.32 ± 0.39	< 0.001	−0.45 to 1.08
Temporal 2 mm	0.00 ± 0.26	0.966	−0.51 to 0.52
Inferior 2 mm	0.14 ± 0.32	< 0.001	−0.48 to 0.77
Nasal 5 mm	−0.01 ± 0.22	0.797	− 0.44 to 0.42
Superior 5 mm	−0.01 ± 0.43	0.876	−0.86 to 0.84
Temporal 5 mm	0.00 ± 0.22	0.875	−0.42 to 0.43
Inferior 5 mm	−0.15 ± 0.27	< 0.001	− 0.68 to 0.37

Keratometric data are in units of diopter (D); Mean ± SD = Mean ± Standard deviation generated by paired T-test; 95% LoA = 95% limits of agreement

## Discussion

In this study, SS-OCT and rotating Scheimpflug camera were comprehensively used to assess the repeatability and reproducibility of CT and refractive power measurements in multiple regions of the cornea in children. The precision (repeatability and reproducibility) evaluation is a mandatory task to test the reliability of new technology, and some findings have been reported in adults previously. Szalai et al. [18] noticed a better repeatability of CCT measurements with CASIA (TRT = 4.17 μm) than with the Pentacam HR (TRT = 7.33 μm), and these values were similar to our study outcomes. The same study reported a contradictory finding wherein CASIA showed a lower repeatability (TRT = 21.922 μm) than Pentacam HR (TRT = 11.451 μm) when measuring TCT. Moreover, the repeatability for keratometry measurements with CASIA (TRT, 0.481–0.555 D) and Pentacam HR (TRT, 0.468–0.472 D) were both lower than that in our study. Neri et al. [23] noticed a better repeatability for CCT measurement using CASIA than that using a spectral-domain OCT (Cirrus-OCT, Carl Zeiss Meditec AG, Germany). Though they listed SD only, we acquired a CoV of 0.33% by calculating the ratio of SD to the corresponding mean value. This value was close to our study results.

Our study showed that the two systems had high precision when measuring both thickness and keratometry metrics in central cornea, with a slight declination towards the periphery. High repeatability for CT and corneal curvature measurements have been reported [7, 24–27]. Xu et al. [28] used Pentacam HR to measure CT and also found a downward trend for precision from central to peripheral region. As explicated before [7], due to the distribution of scan lines in a radial pattern around the visual axis, more points were captured for the central cornea analysis when compared with those at the peripheral region. It was worth noting that the peripheral superior corneal measurements generated the worst precision among all locations with SS-OCT, which was in agreement with the results of our previous study using a spectral-domain OCT to measure CT [7]. The upper eyelashes possibly covered the cornea at this location, and may thus lead to deterioration on the precision of measurement. A similar outcome was found for K measurement using Pentacam HR but the peripheral inferior CT measurement showed the lowest precision. Not surprisingly, the visible blue light that the Pentacam used along with the relatively long scan time could prevent some children from keeping their eyes wide open, making both upper and lower cornea measurement susceptible to the interference of eyelids or eyelashes. However, an unexpected finding was that, apart from the superior positions, the precision for paracentral corneal keratometry measurements with Pentacam HR was lower than that for the peripheral area. A likely explanation for this outcome might be due to the imperfect reconstruction algorithm of Pentacam HR for keratometry measurements in this zone. To the best of our knowledge, this study is the first to evaluate paracentral corneal (the nasal, superior, temporal and inferior position at a distance of 1 mm to the corneal vertex) keratometry.

The precision of astigmatism power vector measurements remained poor with CASIA (repeatability ICC for J_0_, 0.930 to 0.933; ICC for J_45_, 0.715 to 0.724) when compared to Pentacam HR (repeatability ICC for J_0_, 0.961 to 0.962; ICC for J_45_, 0.766 to 0.832). Previous studies [29, 30] reported similar results for Pentacam HR when measuring J_0_ (ICC, 0.974 to 0.979) and J_45_ (ICC, 0.876 to 0.888). These attributed the moderate precision to the small value of corneal astigmatism, and the same explanation could be used for our study. The absolute value of J_0_ derived from the children was greater than that of J_45_, and the measurement of J_0_ showed higher ICC value than J_45_ measurement. Savini et al. [14] used AS-OCT combined with Placido corneal topography (MS-39, Costruzione Strumenti Oftalmici, Florence, Italy) to measure the total corneal astigmatism, and found better repeatability of both J_0_ (ICC = 0.975) and J_45_ (ICC = 0.950) measurements. Taken together, it seems that the Placido disk could improve the astigmatism measurement.

An interesting finding observed in our study was that the SS-OCT outperformed the Scheimpflug-based corneal topographic map in measuring CT, while Scheimpflug-based corneal topographer was observed to be more precise for corneal power measurement. Firstly, the values of repeatable and reproducible CoVs for CT measurements were smaller with CASIA when compared with Pentacam HR. Our previous study compared RTVue and Pentacam in obtaining CT measurements at the same locations as set currently, and the results showed a better repeatability with SD-OCT than with Pentacam [7]. We considered that this disparity might be due to high resolution and short acquisition time of OCT technology. It should be noted that the high-resolution version of Pentacam was employed this time, but higher CoVs for CT measurements were discovered when compared with those reported by our study previously (CoVs, 0.98–2.12% vs. 0.65–1.10%) [7]. A likely reason for this may be related to the lower cooperative degree of children. Despite this, CASIA still generated a slightly higher repeatability than RTVue (CoVs, 0.20–0.75% vs. 0.31–1.16%) when measuring CT [7]. This was probably because the automatic alignment function applied to CASIA minimized the impact resulting from off-axis measurement.

Secondly, the CoV was marginally greater for corneal power measurement with CASIA at each location, indicating better precision with Pentacam HR when acquiring keratometric map of the cornea. This discrepancy was also reported for CASIA and Pentacam HR for the measurements of anterior keratometry on the central cornea [18, 19]. Wang et al. [16] found low precision of keratometry measurement using another SD-OCT (RTVue; repeatable ICC, 0.982–0.990; reproducible TRT, 0.26–0.44 D, reproducible CoV, 0.22–0.36%), as compared with the measurement using CASIA in the current study (repeatable ICC, 0.994–0.995; reproducible TRT, 0.26 D, reproducible CoV, 0.21%). Savini et al. [14] employed SD-OCT combined with Placido device (MS-39) and revealed a higher repeatability of keratometry measurement with a TRT of 0.20 D and a CoV of 0.16%. The simple OCT instrument had a limited role in measuring cornea power, and the combination of OCT and Placido-disk imaging is considered to be an effective means for improving the accuracy. Several studies [11–13] also generated high repeatability of TD-OCT when combined with Placido instrument (Omni, Carl Zeiss Meditec AG, Germany) in measuring corneal power indices.

Though the CT readings provided by CASIA were significantly thinner than those measured by Pentacam HR, the interdevice agreement still remained high for the central cornea. However, the agreement was from moderate to poor for CT measurements from the paracentral to the peripheral region. Milla et al. [26] compared a TD-OCT (Visante) and a Scheimpflug-Placido corneal topographer (Sirius, Costruzione Strumenti Oftalmici, Florence, Italy) when measuring the CCT and peripheral CT. These have set the peripheral CT measurements on a distance of 2.5 mm and 4.0 mm, respectively, to the corneal vertex, and showed smaller CT readings at all locations with OCT instrument, as well as poor agreement between Visante and Sirius with 95% LoA of − 42.7 to − 2.0 μm for CCT measurement, and − 42.8 to 24.0 μm, − 59.1 to 7.6 μm, − 77.5 to 9.2 μm, and − 51.6 to 0.4 μm, respectively for 2.5 mm temporally, nasally, superiorly, and inferiorly, and − 86.1 to − 32.9 μm, − 112.2 to − 22.8 μm, − 88.7 to 15.2 μm, and − 128.9 to 6.2 μm, respectively, for 4.0 mm temporally, nasally, superiorly, and inferiorly. OCT instruments tended to underestimate the CCT values as compared to Scheimpflug-based devices in the normal cornea [11, 26, 31–37], and SS-OCT is not an exception [17–19, 38–41].

With regards to the corneal power measurements, high interdevice agreement was observed for the central cornea. Nakagawa et al. [17] reported lower agreement between the two instruments (95% LoA, − 1.00 to 1.90 D) when measuring the central corneal power, as compared with the current result. A similar outcome has been reported by Szalai et al. [18], which was consistent with our study result. Ghoreishi et al. [38] noticed a high agreement between CASIA and Pentacam HR (95% LoA, − 0.24 to 0.54 D) in adults. As compared with the peripheral regions, agreement for paracentral keratometry measurements was even lower, which resulted from the abnormal precision of Pentacam HR for keratometry measurements in this area.

A limitation of our study would be that only one model of OCT was used. Further investigation is warranted to compare more OCT instruments, including TD-OCT and SD-OCT combined with Placido disk devices. Additionally, investigations are required to determine the precision when enrolling children with abnormal corneas, such as congenital corneal opacities, macrocornea and microcornea.

## Conclusion

In summary, both CASIA SS-OCT and Pentacam high-resolution Scheimpflug system showed high precision when measuring CT and keratometry in children, although a slight decrease in precision was noted for the peripheral cornea. Furthermore, the reliability of CT measurement was higher with the SS-OCT device, while the precision of corneal power measurement was higher with the Scheimpflug imaging system. Therefore, we recommend the use of a pachymetric map of the cornea acquired with AS-OCT and a corneal keratometric map obtained with rotating Scheimpflug camera in clinical practice. In addition, the interdevice agreement of CT measurement was high for the central cornea zone, but moderate for the paracentral and peripheral regions. With respect to measuring corneal power, high agreement was observed when measuring by keratometry in central regions. Hence, only the central and the TCT as well as keratometry in the central area can be used interchangeably between the two devices.

## Supplementary information

**Additional file 1: Table S1.** Interobserver reproducibility outcomes for corneal thickness obtained using CASIA swept-source optical coherence tomography in children.

**Additional file 2: Table S2.** Interobserver reproducibility outcomes for corneal thickness obtained using Pentacam Scheimpflug imaging in children.

**Additional file 3: Table S3.** Interobserver reproducibility outcomes for corneal power obtained using CASIA and swept-source optical coherence tomography in children.

**Additional file 4: Table S4.** Interobserver reproducibility outcomes for corneal power obtained using Pentacam Scheimpflug imaging in children.

## Data Availability

All data generated or analyzed during this study are included in this published article.

## Abbreviations

OCT: Optical coherence tomography
SS-OCT: Swept-source optical coherence tomography
AS-OCT: Anterior-segment optical coherence tomography
SD-OCT: Spectral-domain OCT
K: Keratometry
K_2mm-Nasal_, K_2mm-Superior_, K_2mm-Temporal_, K_2mm-Inferior_: The keratometry at 2 mm diameter in the nasal, superior, temporal and inferior regions centered on the corneal vertex.
K_5mm-Nasal_, K_5mm-Superior_, K_5mm-Temporal_, K_5mm-Inferior_: The keratometry at 5 mm diameter in the nasal, superior, temporal and inferior regions centered on the corneal vertex.
CT: corneal thickness
CT_2mm-Nasal_, CT_2mm-Superior_, CT_2mm-Temporal_, CT_2mm-Inferior_: The corneal thickness at 2 mm diameter in the nasal, superior, temporal and inferior regions centered on the corneal vertex.
CT_5mm-Nasal_, CT_5mm-Superior_, CT_5mm-Temporal_, CT_5mm-Inferior_: The corneal thickness at 5 mm diameter in the nasal, superior, temporal and inferior regions centered on the corneal vertex.
CCT: Central corneal thickness
TCT: Thinnest corneal thickness
Mean ± SD: Mean ± standard deviation
TRT: Test-retest variability
CoV: Coefficient of variation
ICC: Intra-class correlation coefficient
S_w_: Intra-subject standard deviation
LoA: Limit of agreement
