# Supplementary material for: The precision and agreement of corneal thickness and keratometry measurements with SS-OCT versus Scheimpflug imaging
Source: Eye Vis (Lond). 2020 Jun 9;7:32. doi: 10.1186/s40662-020-00197-0 (PMC7285531; doi:10.1186/s40662-020-00197-0)
Supplement: Supplementary file 1 — Additional file 1: Table S1. Interobserver reproducibility outcomes for corneal thickness obtained using CASIA swept-source optical coherence tomography in children. [file 40662_2020_197_MOESM1_ESM.docx]

| **Supplements**  Supp Table 1. Interobserver reproducibility outcomes for corneal thickness obtained using CASIA swept-source optical coherence tomography in children. | | | | |
| --- | --- | --- | --- | --- |
| Parameter | S_w_ | TRT | CoV (%) | ICC (95% CI) |
| Center | 0.95 | 2.63 | 0.18 | 0.999 (0.998 to 0.999) |
| Thinnest | 1.20 | 3.32 | 0.23 | 0.998 (0.997 to 0.999) |
| Nasal 2mm | 1.69 | 4.68 | 0.31 | 0.997 (0.995 to 0.998) |
| Superior 2mm | 1.50 | 4.16 | 0.27 | 0.997 (0.996 to 0.998) |
| Temporal 2mm | 1.31 | 3.62 | 0.24 | 0.998 (0.997 to 0.999) |
| Inferior 2mm | 1.44 | 4.00 | 0.27 | 0.998 (0.996 to 0.999) |
| Nasal 5mm | 2.82 | 7.80 | 0.48 | 0.991 (0.986 to 0.994) |
| Superior 5mm | 2.83 | 7.85 | 0.48 | 0.991 (0.987 to 0.995) |
| Temporal 5mm | 2.15 | 5.96 | 0.38 | 0.995 (0.992 to 0.997) |
| Inferior 5mm | 2.39 | 6.62 | 0.42 | 0.994 (0.991 to 0.996) |
| Thickness data are in units of micrometer (μm); SD = standard deviation, S_w_ = within-subject standard deviation, TRT = test-retest repeatability (2.77 S_w_), CoV = within-subject coefficient of variation, ICC = intraclass correlation coefficient. | | | | |
