# Supplementary material for: The precision and agreement of corneal thickness and keratometry measurements with SS-OCT versus Scheimpflug imaging
Source: Eye Vis (Lond). 2020 Jun 9;7:32. doi: 10.1186/s40662-020-00197-0 (PMC7285531; doi:10.1186/s40662-020-00197-0)
Supplement: Supplementary file 2 — Additional file 2: Table S2. Interobserver reproducibility outcomes for corneal thickness obtained using Pentacam Scheimpflug imaging in children. [file 40662_2020_197_MOESM2_ESM.docx]

| Supp Table 2. Interobserver reproducibility outcomes for corneal thickness obtained using Pentacam Scheimpflug imaging in children. | | | | |
| --- | --- | --- | --- | --- |
| Parameter | S_w_ | TRT | CoV (%) | ICC (95% CI) |
| Center | 3.61 | 10.01 | 0.67 | 0.984 (0.975 to 0.990) |
| Thinnest | 4.02 | 11.14 | 0.75 | 0.980 (0.970 to 0.987) |
| Nasal 2mm | 4.34 | 12.02 | 0.78 | 0.977 (0.965 to 0.985) |
| Superior 2mm | 4.37 | 12.11 | 0.78 | 0.977 (0.964 to 0.985) |
| Temporal 2mm | 4.10 | 11.36 | 0.76 | 0.980 (0.968 to 0.987) |
| Inferior 2mm | 4.43 | 12.26 | 0.81 | 0.976 (0.963 to 0.985) |
| Nasal 5mm | 7.56 | 20.94 | 1.24 | 0.941 (0.909 to 0.962) |
| Superior 5mm | 6.88 | 19.06 | 1.10 | 0.954 (0.929 to 0.970) |
| Temporal 5mm | 5.86 | 16.23 | 1.01 | 0.963 (0.943 to 0.976) |
| Inferior 5mm | 7.61 | 21.08 | 1.31 | 0.941 (0.910 to 0.962) |
| Thickness data are in units of micrometer (μm); SD = standard deviation, S_w_ = within-subject standard deviation, TRT = test-retest repeatability (2.77 S_w_), CoV = within-subject coefficient of variation, ICC = intraclass correlation coefficient. | | | | |
