# Supplementary material for: The precision and agreement of corneal thickness and keratometry measurements with SS-OCT versus Scheimpflug imaging
Source: Eye Vis (Lond). 2020 Jun 9;7:32. doi: 10.1186/s40662-020-00197-0 (PMC7285531; doi:10.1186/s40662-020-00197-0)
Supplement: Supplementary file 3 — Additional file 3: Table S3. Interobserver reproducibility outcomes for corneal power obtained using CASIA and swept-source optical coherence tomography in children. [file 40662_2020_197_MOESM3_ESM.docx]

| Supp Table 3. Interobserver reproducibility outcomes for corneal power obtained using CASIA and swept-source optical coherence tomography in children. | | | | |
| --- | --- | --- | --- | --- |
| Parameter | S_w_ | TRT | CoV (%) | ICC (95% CI) |
| Km | 0.09 | 0.26 | 0.21 | 0.996 (0.993 to 0.997) |
| J_0_ | 0.09 | 0.25 | - | 0.958 (0.934 to 0.973) |
| J_45_ | 0.10 | 0.28 | - | 0.784 (0.681 to 0.857) |
| Nasal 2mm | 0.15 | 0.41 | 0.35 | 0.988 (0.982 to 0.992) |
| Superior 2mm | 0.22 | 0.60 | 0.49 | 0.983 (0.973 to 0.989) |
| Temporal 2mm | 0.17 | 0.47 | 0.39 | 0.985 (0.977 to 0.990) |
| Inferior 2mm | 0.18 | 0.51 | 0.42 | 0.987 (0.980 to 0.992) |
| Nasal 5mm | 0.19 | 0.52 | 0.44 | 0.982 (0.972 to 0.988) |
| Superior 5mm | 0.26 | 0.72 | 0.60 | 0.976 (0.963 to 0.985) |
| Temporal 5mm | 0.18 | 0.50 | 0.42 | 0.983 (0.973 to 0.989) |
| Inferior 5mm | 0.20 | 0.55 | 0.46 | 0.984 (0.976 to 0.990) |
| Keratometric data are in units of diopter (D); SD = standard deviation, S_w_ = within-subject standard deviation, TRT = test-retest repeatability (2.77 S_w_), CoV = within-subject coefficient of variation, ICC = intraclass correlation coefficient. | | | | |
