# Supplementary material for: The precision and agreement of corneal thickness and keratometry measurements with SS-OCT versus Scheimpflug imaging
Source: Eye Vis (Lond). 2020 Jun 9;7:32. doi: 10.1186/s40662-020-00197-0 (PMC7285531; doi:10.1186/s40662-020-00197-0)
Supplement: Supplementary file 4 — Additional file 4: Table S4. Interobserver reproducibility outcomes for corneal power obtained using Pentacam Scheimpflug imaging in children. [file 40662_2020_197_MOESM4_ESM.docx]

| Supp Table 4. Interobserver reproducibility outcomes for corneal power obtained using Pentacam Scheimpflug imaging in children. | | | | |
| --- | --- | --- | --- | --- |
| Parameter | S_w_ | TRT | CoV (%) | ICC (95% CI) |
| Km | 0.05 | 0.14 | 0.12 | 0.999 (0.998 to 0.999) |
| J_0_ | 0.05 | 0.14 | - | 0.988 (0.981 to 0.992) |
| J_45_ | 0.06 | 0.16 | - | 0.931 (0.894 to 0.955) |
| Nasal 2mm | 0.11 | 0.31 | 0.27 | 0.993 (0.990 to 0.996) |
| Superior 2mm | 0.12 | 0.33 | 0.27 | 0.995 (0.992 to 0.997) |
| Temporal 2mm | 0.12 | 0.32 | 0.27 | 0.993 (0.989 to 0.995) |
| Inferior 2mm | 0.14 | 0.38 | 0.31 | 0.993 (0.990 to 0.996) |
| Nasal 5mm | 0.07 | 0.20 | 0.17 | 0.997 (0.996 to 0.998) |
| Superior 5mm | 0.15 | 0.41 | 0.34 | 0.991 (0.986 to 0.994) |
| Temporal 5mm | 0.06 | 0.16 | 0.14 | 0.998 (0.997 to 0.999) |
| Inferior 5mm | 0.07 | 0.21 | 0.17 | 0.998 (0.996 to 0.999) |
| Keratometric data are in units of diopter (D); SD = standard deviation, S_w_ = within-subject standard deviation, TRT = test-retest repeatability (2.77 S_w_), CoV = within-subject coefficient of variation, ICC = intraclass correlation coefficient. | | | | |
